# Correlation between the triglyceride-glucose index and chronic kidney disease among adults with metabolic-associated fatty liver disease: fourteen-year follow-up

**DOI:** 10.3389/fendo.2024.1400448

**Published:** 2024-05-23

**Authors:** Suosu Wei, Tengyan Wu, Yanwu You, Fei Liu, Qiyan Hou, Chongde Mo, Lei Zhou, Jianrong Yang

**Affiliations:** ^1^ Department of Scientific Cooperation of Guangxi Academy of Medical Sciences, People’s Hospital of Guangxi Zhuang Autonomous Region, Nanning, China; ^2^ Department of Health Service Management, School of Information and Management, Guangxi Medical University, Nanning, China; ^3^ Department of Nephrology, People’s Hospital of Guangxi Zhuang Autonomous Region, Nanning, China; ^4^ Scientific Research and Experimental Center, The People’s Hospital of Guangxi Zhuang Autonomous Region, Guangxi Academy of Medical Sciences, Nanning, China; ^5^ Graduate School of Guangxi University of Chinese Medicine, Nanning, China; ^6^ Department of Colorectal and Anal Surgery, Guangxi Academy of Medical Sciences, People’s Hospital of Guangxi Zhuang Autonomous Region, Nanning, China; ^7^ Guangxi Academy of Medical Sciences, The People’s Hospital of Guangxi Zhuang Autonomous Region, Nanning, China; ^8^ Department of Hepatobiliary, Pancreas and Spleen Surgery, Guangxi Academy of Medical Sciences, People’s Hospital of Guangxi Zhuang Autonomous Region, Nanning, China

**Keywords:** triglyceride-glucose, cohort study, chronic kidney disease, metabolic dysfunction-associated fatty liver disease, incidence rate

## Abstract

**Background and aims:**

According to previous studies, triglyceride-glucose (TyG) is related to chronic kidney disease (CKD), but no studies have explored the correlation between TyG and CKD among adults with metabolic dysfunction-associated fatty liver disease (MAFLD). We aimed to explore the associations of the TyG index with CKD among adults with MAFLD.

**Methods:**

In this retrospective observational cohort study, data from 11,860 participants who underwent a minimum of three health assessments between 2008 and 2015 were retrospectively collected. Participants were followed up until the final medical visit or health examination. CKD refers to an eGFR < 60 mL/min per 1·73 m^2^ or the occurrence of two or more incidents of proteinuria.

**Results:**

Within a median 10·02-year follow-up period, 2005 (16·9%) participants reported developing CKD. Multivariate Cox regression models indicated a noticeable correlation between the TyG index and CKD incidence (HR per unit increase, 1.19; 95% CI: 1.09–1.29) and between the TyG index and CKD incidence (HR per SD increase, 1.12; 95% CI: 1.06–1.18). The CKD incidence increased by 1.8 times in participants in the highest TyG index quartile relative to patients in the lowest quartile of the TyG index quartile (HR 1·18, 95% CI: 1.01–1.38, P = 0.007). According to subgroup analysis, an elevated TyG index is likely to become more harmful to participants younger than 60 years (P for interaction = 0.035).

**Conclusion:**

An elevated TyG index may increase CKD incidence among MAFLD adults, particularly among younger people. Early intervention may help reduce the incidence of CKD.

## 1 Introduction

Chronic kidney disease (CKD) is a leading global health concern, contributing to morbidity and mortality worldwide and representing the main risk factor related to cardiovascular disease. Additionally, the incidence of CKD is expected to increase with the aging population and the widespread prevalence of hypertension and diabetes mellitus (DM). CKD has an increasing prevalence due to population aging and the extensive incidence of DM and hypertension. According to a recent study, the global all-age mortality rate from CKD increased by 41.5% between 1990 and 2017, resulting in an estimated 1.2 million deaths worldwide (1). CKD is often asymptomatic until it reaches its advanced stages, but it is a chronic condition that can be prevented and treated. Identifying high-risk CKD patients early and providing timely interventions can help lower the incidence and mortality of CKD.

In 2020, an international panel of experts recommended altering nonalcoholic fatty liver disease (NAFLD) terminology to metabolic dysfunction-related fatty liver disease (MAFLD) due to the difficulty of applying its definition in clinical practice (2, 3). Patients with MAFLD have a greater proportion of metabolic comorbidities than NAFLD patients (4). Furthermore, patients with MAFLD have greater odds of developing severe extrahepatic organ disease, chronic cardiovascular disease, CKD, and even all-cause mortality than NAFLD patients (5). These findings suggest that MAFLD and NAFLD patients have significant clinical differences. Additionally, individuals with MAFLD have an increased risk of CKD compared with individuals without MAFLD (6). Therefore, it is vital to classify individuals with a greater risk of CKD in the MAFLD population and to provide timely interventions to reduce CKD risk.

As an early metabolic change in CKD patients, insulin resistance can contribute to traditional risk factors and work synergistically with kidney-specific mechanisms to cause CKD (7). Typically, the triglyceride-glucose (TyG) index is an easy and reliable measure of IR. It is measured by multiplying fasting triglyceride (TG) levels with fasting blood glucose (FBG) levels. It can be used to detect IR in an easy and inexpensive way relative to the homeostasis model assessment of insulin resistance (HOMA-IR) index (8). Studies have indicated that an elevated TyG index increases CKD incidence among healthy individuals, patients with diabetes, patients with coronary atherosclerotic heart disease, and patients on peritoneal dialysis (9–15). Nevertheless, the association between the TyG index and CKD among MAFLD patients is unclear. Therefore, the current work aimed to study the relationship between the TyG index and CKD incidence among MAFLD patients.

## 2 Methods

### 2.1 Study design and population

The materials in this work were part of an ambispective cohort study (Registration site: http://www.chictr.org.cn/index.aspx; registration number: ChiCTR2200058543) using an active health management platform described earlier (16). This platform links and indices all data from hospital visits, including outpatient, inpatient, and physical examination data. The medical information generated during a patient visit can be automatically integrated into this platform. This cohort (n = 49,719) consisted of participants who underwent three or more physical examinations at the People’s Hospital of the Guangxi Zhuang Autonomous Region, China, during 2008–2015. The initial health examination date was used as the index date. In addition, the following exclusion criteria were used for adult participants: 1) unavailable demographic data, 2) a basic estimated glomerular filtration rate (eGFR) <60 mL/min every 1·73 m^2^ or more severe proteinuria, 3) unavailable laboratory test results concerning MAFLD diagnosis, and 4) a follow-up time < 1 year. In this study, data from 11,860 participants were analyzed (**Supplementary Figure S1**).

### 2.2 MAFLD confirmation

This work used ultrasound for fatty liver diagnosis according to Asia-Pacific guidelines (17). MAFLD was confirmed in patients with fatty liver and the following conditions: 1) overweight/obesity (body mass index [BMI] ≥ 23 kg/m^2^), 2) type 2 DM (T2MD), and 3) lean/normal weight (BMI <23 kg/m^2^) and the presence of two or more metabolic abnormalities (3).

### 2.3 Data extraction

We collected baseline data for participants at the index date. When any data were not available on the index date, we imported data near this date. Additionally, we used the eGFR equation developed by the Chronic Kidney Disease Epidemiology Collaboration as a measure of renal function (18). This study assessed covariates such as underlying BMI, hypertension, T2DM, and dyslipidemia. We classified BMI as lean/normal weight (<23) or overweight/obese (≥23) (3). In addition, diastolic blood pressure ≥ 90 mmHg or systolic blood pressure ≥140 mmHg were considered the diagnostic criteria for hypertension. Additionally, HbA1c ≥ 6·5 or FBG ≥7·0 mmol/L indicated T2DM. In addition, TG ≥ 1·7 mmol/L or low-density lipoprotein (LDL) cholesterol ≥ 2·6 mmol/L indicated dyslipidemia. In addition, diagnostic procedures and laboratory tests were performed at the health examination hospital laboratory.

### 2.4 Study outcomes

We followed up on patients who experienced CKD or proteinuria during the time of enrollment until the final medical visit or health examination. In addition, censored events can be considered an inability to follow up and study termination. Our primary endpoints included CKD, which was defined as an eGFR < 60 mL/min/1·73 m^2,^ and proteinuria (at least two) detected by the dipstick approach during the follow-up period.

### 2.5 Statistical analysis

Continuous data are expressed as medians and interquartile ranges (IQRs) and were analyzed via ANOVA or the Kruskal−Wallis test, whereas categorical data are expressed as numbers and percentages and were analyzed via the chi−square test. According to the TyG index quartiles, we classified the participants into four groups.

The incidence of CKD was calculated by dividing the number of cases among the population within the follow-up period by the person-year number of follow-ups. The cumulative CKD incidence of different TyG groups was analyzed by Kaplan−Meier analysis, and group comparisons were performed with the log-rank test. The Cox hazard model was adopted for identifying hazard ratios (HRs) and 95% confidence intervals (CIs) for the risk of CKD in patients with TyG syndrome, and the proportional hazards assumption of the Cox model was assessed using Schoenfeld residuals. Based on the backward selection method and literature review, covariates were selected, and additional possible confounding factors were detected (11, 13). The covariates in Model 1 remained nonadjusted. Model 2 was adjusted for age, sex, hypertension status, T2DM status, dyslipidemia status, and BMI, while Model 3 was adjusted for total cholesterol (TC), LDL, aspartate aminotransferase (AST), alanine aminotransferase (ALT), and baseline eGFR based on Model 2. Additionally, we adopted restricted cubic spline (RCS) analysis to evaluate the dose−response correlation of the TyG index with CKD.

Finally, several subgroup analyses were conducted with prespecified subgroups. R software (version 3.3.2) and SPSS 18 were used for the statistical analysis. *P* < 0.05 (two-tailed) indicated statistical significance.

## 3 Results

### 3.1 Baseline characteristics

The median [IQR] age of the 11,860 MAFLD patients included in the analysis was 45·10 [37·00–53·00], and 76·4% of them were male. In addition, for the baseline TyG index, the median [IQR] was 8·96 (8·59; 9·39). We found that participants with an increased TyG index exhibited increased blood pressure (BP), ALT, AST, waist circumference (WC), TC, FBG, TG, and LDL levels compared with those with a lower TyG index. Men who exhibited hypertension, dyslipidemia, overweight, or type 2 diabetes had increased TyG index values (**Table 1**).

**Table 1:** Baseline characteristics of participants according to quartiles of TyG index.

Characteristic	Overall	Q1(7.08-8.59)	Q2 (8.60-8.96)	Q3(8.97-9.39)	Q4(9.40-12.95)	P-value
	(n=11860)	(n=2964)	(n=2966)	(n=2964)	(n=2966)	
Demographic parameters						
Age, median (IQR), y	45.00 (37.00-53.00)	45.00 (36.00-53.00)	45.00 (36.00-53.00)	45.00 (37.00-53.00)	45.00 (38.00-52.00)	0.184
Sex/female	2798 (23.59)	934 (31.5)	760 (25.6)	623 (21.0)	481 (16.2)	
Sex/male	9062 (76.41)	2030 (68.5)	2206 (74.4)	2341 (79.0)	2485 (83.8)	
BMI, median (IQR), kg/m^2^	26.71 (25.05-28.63)	26.59 (24.98-28.57)	26.72 (25.12-28.56)	26.82 (25.04-28.71)	26.75 (25.04-28.67)	0.635
Waist circumference, median (IQR), cm	93.00 (88.00-98.00)	92.00 (87.00-97.00)	92.00 (88.00-97.75)	93.00 (88.00-98.00)	93.50 (88.00-98.00)	<0.001
Blood pressure, median (IQR), mmHg						
Systolic	129.00 (119.00-140.00)	127.00 (117.00-138.00)	128.00 (119.00-139.00)	130.00 (120.00-140.00)	132.00 (122.00-143.00)	<0.001
Diastolic	80.00 (73.00-87.00)	77.00 (70.00-85.00)	79.00 (72.00-87.00)	80.00 (73.00-88.00)	82.00 (75.00-90.00)	<0.001
Laboratory parameter, median (IQR)						
Fasting glucose, mmol/L	5.31 (4.91-5.82)	5.09 (4.73-5.47)	5.25 (4.86-5.66)	5.33 (4.95-5.82)	5.71 (5.22-6.49)	<0.001
ALT, U/L	26.00 (19.00-38.00)	23.00 (17.00-31.00)	26.00 (19.00-37.00)	28.00 (20.00-39.00)	32.00 (23.00-46.00)	<0.001
AST, U/L	23.00 (20.00-28.00)	22.00 (18.00-26.00)	23.00 (19.00-28.00)	24.00 (20.00-29.00)	25.00 (21.00-31.00)	<0.001
TC, mmol/L	5.27 (4.67-5.91)	4.88 (4.34-5.50)	5.22 (4.67-5.80)	5.40 (4.82-6.03)	5.59 (4.97-6.30)	<0.001
HDL, mmol/L	1.16 (0.99-1.35)	1.30 (1.14-1.50)	1.20 (1.06-1.37)	1.13 (0.99-1.30)	0.99 (0.86-1.13)	<0.001
TG, mmol/L	1.82 (1.27-2.70)	1.01 (0.83-1.17)	1.55 (1.39-1.72)	2.21 (1.96-2.51)	3.64 (3.01-4.97)	<0.001
LDL, mmol/L	3.44 (2.99-4.00)	3.20 (2.77-3.71)	3.49 (3.06-4.01)	3.59 (3.18-4.15)	3.50 (2.97-4.11)	<0.001
eGFR, mL/min/1·73m^2^	96.99 (86.11-106.59)	97.66 (86.65-107.72)	96.14 (84.97-105.82)	96.65 (85.86-105.67)	97.67 (87.25-107.10)	<0.001
TyG index	8.96 (8.59-9.39)	8.33 (8.14-8.48)	8.78 (8.69-8.87)	9.16 (9.06-9.27)	9.72 (9.53-10.06)	<0.001
Comorbidities						
Hypertension	3665 (30.90)	771 (26.0)	858 (28.9)	926 (31.2)	1110 (37.4)	<0.001
Dyslipidemia	9110 (76.81)	1410 (47.6)	1903 (64.2)	2850 (96.2)	2947 (99.4)	<0.001
Overweight	11362 (95.80)	2895 (97.7)	2868 (96.7)	2806 (94.7)	2793 (94.2)	<0.001
Type 2 diabetes	2234 (18.84)	240 (8.1)	398 (13.4)	560 (18.9)	1036 (34.9)	<0.001

Values are expressed as number (percentage), unless indicated otherwise.

IQR, Inter Quartile Range; BMI, body mass index; ALT, alanine aminotransferase; AST, aspartate transaminase; TC, total cholesterol; HDL, high-density lipoprotein; LDL, low-density lipoprotein; TG, triglyceride; TyG, triglyceride–glucose.

### 3.2 Correlation between the TyG index and CKD incidence

**Figure 1:**
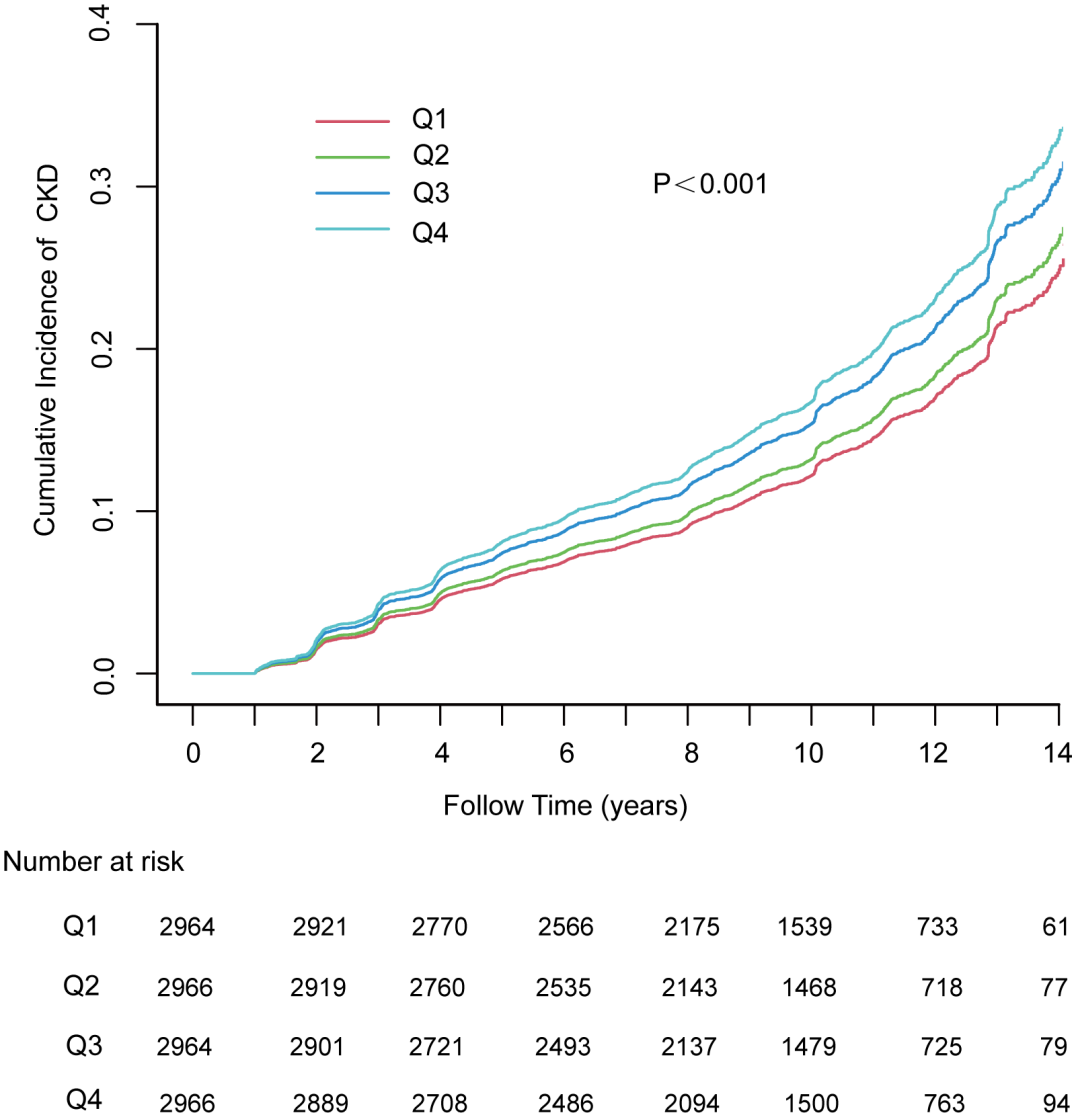
The cumulative incidence of chronic kidney disease based on quartiles of TyG index; P-value was generated based on log-rank test. MAFLD, metabolic dysfunction-associated fatty liver disease; CKD, chronic kidney disease.

Within the 10.02-year (IQR: 7·56–11·99) median follow-up period, the person-year number was 112,726·06, while CKD was reported among 2005 (16·9%) participants (177·86/10,000 person-years; 95% CI, 170·16–185·82). In addition, the incidence of CKD tended to increase with increasing TyG index quartiles from 148·64/10,000 person-years (95% CI, 134·82–163·47) in quartile 1 to 211·35/10,000 person-years (95% CI, 194·66–299·08) in quartile 4. In addition, the risk of CKD among the participants within the fourth quartile of the TyG index increased relative to that in the other quartiles (log-rank test, P < 0.001; **Figure 1**).

**Figure 2:**
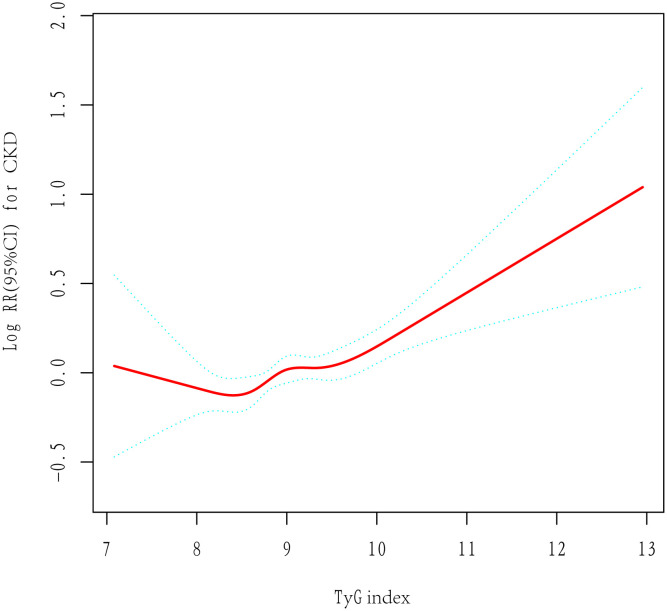
The adjusted restricted cubic spline of the association between the triglyceride–glucose index and incident chronic kidney disease. Adjusted for age, sex, hypertension, type 2 diabetes, dyslipidemia, BMI, TC, LDL, ALT, AST and baseline eGFR.

**Table 2** presents the findings from the Cox regression models. According to unadjusted Cox regression, an increased TyG index was a risk factor related to CKD. Additionally, a positive association existed even after adjustment for various confounders in Model 1 and Model 2. The risk of developing CKD increased by 19% (95% CI, 1·09–1·29, p<0.001) per unit increase in the TyG index. In addition, with the lowest quartile serving as a reference, the adjusted HRs (95% CIs) were shown to be 1·02 (0·89, 1·16), 1·13 (0·98, 1·31), and 1·18 (1·01, 1·38) within the second, third and highest TyG index quartiles, respectively. The results were found to be similar for each 1 standard deviation (SD) increment. Linear trend analysis revealed a gradual increase in the risk of incident CKD with increasing TyG index quartiles, regardless of adjustment (P <0·001). Additionally, a linear correlation between the TyG index and CKD risk existed in the adjusted RCS (P nonlinearity = 0·067) (**Figure 2**).

**Table 2:** Association between triglyceride-glucose index and incident chronic kidney disease.

Variable	CKD Event (%)	Mode l		Model 2		Model 3	
		HR (95% CI)	*P*–value	HR (95% CI)	*P*–value	HR (95% CI)	*P*–value
TyG	2005(16.9)	1.26 (1.18, 1.34)	<0.001	1.16 (1.07, 1.26)	<0.001	1.19 (1.09, 1.29)	<0.001
TyG per 1SD		1.16 (1.12, 1.21)	<0.001	1.10 (1.05, 1.16)	<0.001	1.12 (1.06, 1.18)	<0.001
TyG quartiles							
Q1	424(14.3)	Reference		Reference		Reference	
Q2	456(15.4)	1.09 (0.95, 1.24)	0.207	1.05 (0.92, 1.20)	0.49	1.02 (0.89, 1.16)	0.818
Q3	533(18.0)	1.28 (1.13, 1.46)	<0.001	1.16 (1.00, 1.34)	0.044	1.13 (0.98, 1.31)	0.098
Q4	592(20.0)	1.41 (1.24, 1.60)	<0.001	1.20 (1.03, 1.40)	0.018	1.18 (1.01, 1.38)	0.032
*P* for trend		<0.001		<0.001		<0.001	

Model 1 is not adjusted for any of the variables.

Model 2 adjusted for age, sex, hypertension, type 2 diabetes, dyslipidemia and BMI.

Model 3 adjusted for all the variables in model 1, plus TC, LDL, ALT, AST and baseline eGFR.

BMI, body mass index; ALT, alanine aminotransferase; AST, aspartate transaminase; TC, total cholesterol; LDL, low-density lipoprotein; TyG, triglyceride–glucose.

### 3.3 Subgroup analysis

**Figure 3:**
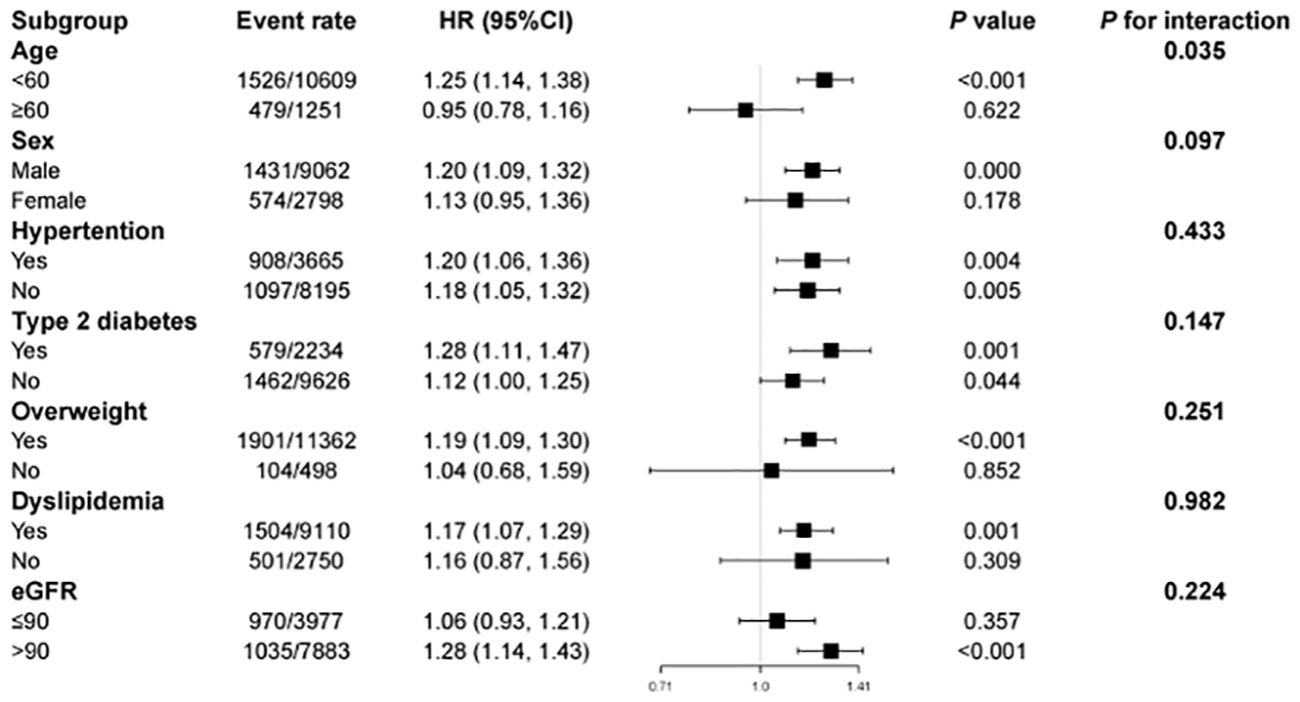
Subgroup analysis of the association between the triglyceride–glucose index and incident chronic kidney disease. HR, hazard ratio; CI, confidence interval. Each stratification was adjusted for all the factors (age, sex, hypertension, type 2 diabetes, dyslipidemia, BMI, TC, LDL, ALT, AST and baseline eGFR) except the stratification factor itself.

The correlations of the TyG index with CKD risk among different subgroups showed that after confounders were adjusted, the TyG index was positively correlated with CKD incidence, which was consistent with age, sex, BMI, and baseline eGFR, as well as with hypertension, type 2 diabetes, overweight status, or dyslipidemia. In contrast, an increased TyG index appeared to be more hazardous for those under the age of 60 years (P for interaction = 0·035) (**Figure 3**).

## 4 Discussion

Our longitudinal cohort study revealed a significant independent relationship between the TyG index and CKD in patients with MAFLD. Additionally, the TyG index demonstrated a positive relationship with CKD risk, whether within quartiles or as a continuous variable. Interestingly, subgroup analysis revealed that an increased TyG index appeared to be more dangerous for those under the age of 60 years. Hence, early intervention is crucial for preventing CKD among patients with MAFLD.

Consistent with previous findings, the incidence of CKD was found to be 1.18-fold greater among MAFLD patients in the highest TyG index quartile than among those in the lowest quartile. A cohort study from China comprising 2,033 participants experiencing glucose metabolic disorder and hypertension reported that relative to people in the lowest TYG index quartile, individuals within the highest TYG index quartile exhibited greater CKD incidence [HR 1·63 (95% CI, 1·14–2·33)] (11). Another cohort study with 7,822 elderly participants (≥ 65 years) in southern China revealed that those within Q4 had an increased CKD incidence relative to those within Q1 [HR 1·59 (95% CI, 1·35–1·88)] (13). The TyG index revealed a significant risk of CKD incidence in both male [HR 1·32 (95% CI, 1·02–1·70)] and female populations [HR 1·50 (95% CI, 1·05–2·13)], as reported in a cohort study of a Japanese medical examination population comprising a median follow-up of four years (14). According to a cohort study conducted on diabetic patients from Singapore, each unit increase in TyG showed a correlation with a 21% (HR 1·21 [95% CI, 1·06–1·37]) increase in CKD incidence (12). Overall, the findings suggest that participants with higher TyG indices are more likely to develop CKD, particularly those exhibiting metabolic risk profiles and hypertension.

The HOMA-IR score is extensively applied to estimate IR, and it can be determined from insulin and FBG levels. Nonetheless, in clinical settings, insulin is not routinely measured, making it impossible to compute HOMA-IR (19). Hence, the TyG index is applied as an alternative IR marker. TyG has been shown to correlate well with both the HOMA-IR index and the normoglycemic-hyperinsulin clamp test (20). Consequently, the TyG index may be used for assessing IR in nations with limited primary care resources. In addition, the mechanism underlying the association between the TyG index and CKD remains unknown. By examining the association of IR with CKD, it is possible to better understand how the TyG index correlates with CKD. IR is a significant risk factor for developing CKD (7, 21). With the occurrence of IR, glucose utilization from adipocytes becomes impaired, leading to aberrant lipid profiles and fasting glucose. Hyperglycemia caused by IR may further result in hypoinflammation and fibrosis (22). IR can also contribute to glomerular hyperfiltration, increased sodium retention, and tubular dysfunction, including inflammation and fibrosis of the renal tissue (23). Hyperinsulinemia is reported to increase sodium reabsorption and renin-angiotensin system activity, cause renal vasodilation, and induce glomerular hyperfiltration, thereby increasing the eGFR (24). An increase in the eGFR per unit leads to glomerular hypertension and renal unit loss, which in turn causes glomerulosclerosis as well as renal failure (25). IR is associated with CKD since it is accompanied by inflammation, oxidative stress, and metabolic acidosis. Moreover, CKD is interrelated with inflammation (26). IR impairs the insulin pathway and elevates monocyte chemotactic protein-1 generation to damage adipose tissue (27). Signals from dysfunctional adipose tissue activate M2 macrophages, thereby releasing proinflammatory cytokines such as interleukin 6 (IL-6) and tumor necrosis factor-alpha (TNFα) (28). IL-6 can contribute to vascular hypertrophy and endothelial dysfunction caused by angiotensin II (29). Similarly, TNFα can lead to endothelial dysfunction (30), which is related to CKD development (31). Nuclear factor red lineage-2 related factor-2 (Nrf2) activation can protect kidneys from oxidative damage (32). Hyperglycemia-induced metabolic acidosis may result in IR (33). The excess acid load generated by metabolic acidosis results in altered renal function, with increased renal plasma flow and GFR (34). These studies indicate a possible mechanism related to the correlation between the TyG index and CKD; however, further research is needed to validate the findings.

The current work has the following strengths: This is the first longitudinal cohort study design for examining the relationship between the TyG index and CKD among patients with MAFLD. Furthermore, a population-based cohort study revealed a high risk of developing CKD. The findings may also help prevent and treat CKD. Additionally, this study had a longer follow-up duration and greater confidence in the findings than other similar cohort studies. However, this study has potential limitations. First, there is a possibility of selection bias given that the participants were urban residents who consistently received annual health examinations at a single-center hospital. Second, ultrasound was used instead of invasive liver biopsy to identify fatty liver. However, in a large-scale epidemiological study, ultrasound, which has high sensitivity and specificity, was used for fatty liver diagnosis (35). Third, this study did not use a quantitative test to measure proteinuria but rather assessed kidney function based on participants’ annual health checkups. Therefore, some participants with acute kidney injury may have been diagnosed with chronic kidney failure (CKD). In addition, the diagnostic criteria also change over time, such as a diabetes diagnosis requiring two abnormal glucose or glycated hemoglobin measurements. Therefore, misclassification bias exists. However, most people’s diagnoses are confirmed by a specialist. Fourth, although this study focused on adjusting for potential risk factors for CKD, other unmeasured or residual confounding factors, including high-sensitivity C-reactive protein levels, medication status for diabetes and hypertension, etc., were not adequately adjusted. Therefore, the impact of these factors on the outcome cannot be evaluated. Nevertheless, the results remain robust and reliable due to the high homogeneity of the patients and the long follow-up time. Fifth, this study did not take into account the severity of liver steatosis. Sixth, because the abnormal metabolic indicators of participants vary from year to year, the metabolic profile of the participants in this study is not always indicative of their true levels based merely on baseline metabolic status. Therefore, future studies are required to examine how the dynamics of the participants’ metabolic indicators are related to the outcome. Finally, the participants were a middle-aged Asian cohort who received annual health examinations; therefore, the results may not be generalizable to groups of different ages, ethnicities, or comorbidities.

## 5 Conclusions

A higher TyG index is positively related to a greater risk of developing CKD. Additionally, more focus needs to be placed on early intervention in MAFLD patients with elevated TyG indices to prevent the progression of CKD and, thus lower the incidence of premature death.

## Data availability statement

The raw data supporting the conclusions of this article will be made available by the authors, without undue reservation.

## Ethics statement

The studies involving humans were approved by Ethics Committee of People’s Hospital of Guangxi Zhuang Autonomous Region, China (LL-KY-QT-202203). The studies were conducted in accordance with the local legislation and institutional requirements. The ethics committee/institutional review board waived the requirement of written informed consent for participation from the participants or the participants' legal guardians/next of kin because This study did not require informed consent as it used retrospectively collected anonymized data.

## Author contributions

SW: Conceptualization, Investigation, Methodology, Supervision, Writing – original draft, Writing – review & editing. TW: Writing – original draft, Writing – review & editing. YY: Data curation, Formal Analysis, Investigation, Writing – review & editing. FL: Conceptualization, Methodology, Writing – review & editing. QH: Conceptualization, Methodology, Writing – review & editing. CM: Conceptualization, Investigation, Methodology, Supervision, Writing – review & editing. LZ: Conceptualization, Methodology, Supervision, Writing – review & editing. JY: Conceptualization, Formal Analysis, Investigation, Methodology, Supervision, Writing – review & editing.

## Supplementary material

Supplementary Figure S1 Flow chart for the selection of participants.
