# Supplementary figures and images for: Correlation between the triglyceride-glucose index and chronic kidney disease among adults with metabolic-associated fatty liver disease: fourteen-year follow-up
Source: Front Endocrinol (Lausanne). 2024 May 23;15:1400448. doi: 10.3389/fendo.2024.1400448 (PMC11153799; doi:10.3389/fendo.2024.1400448)

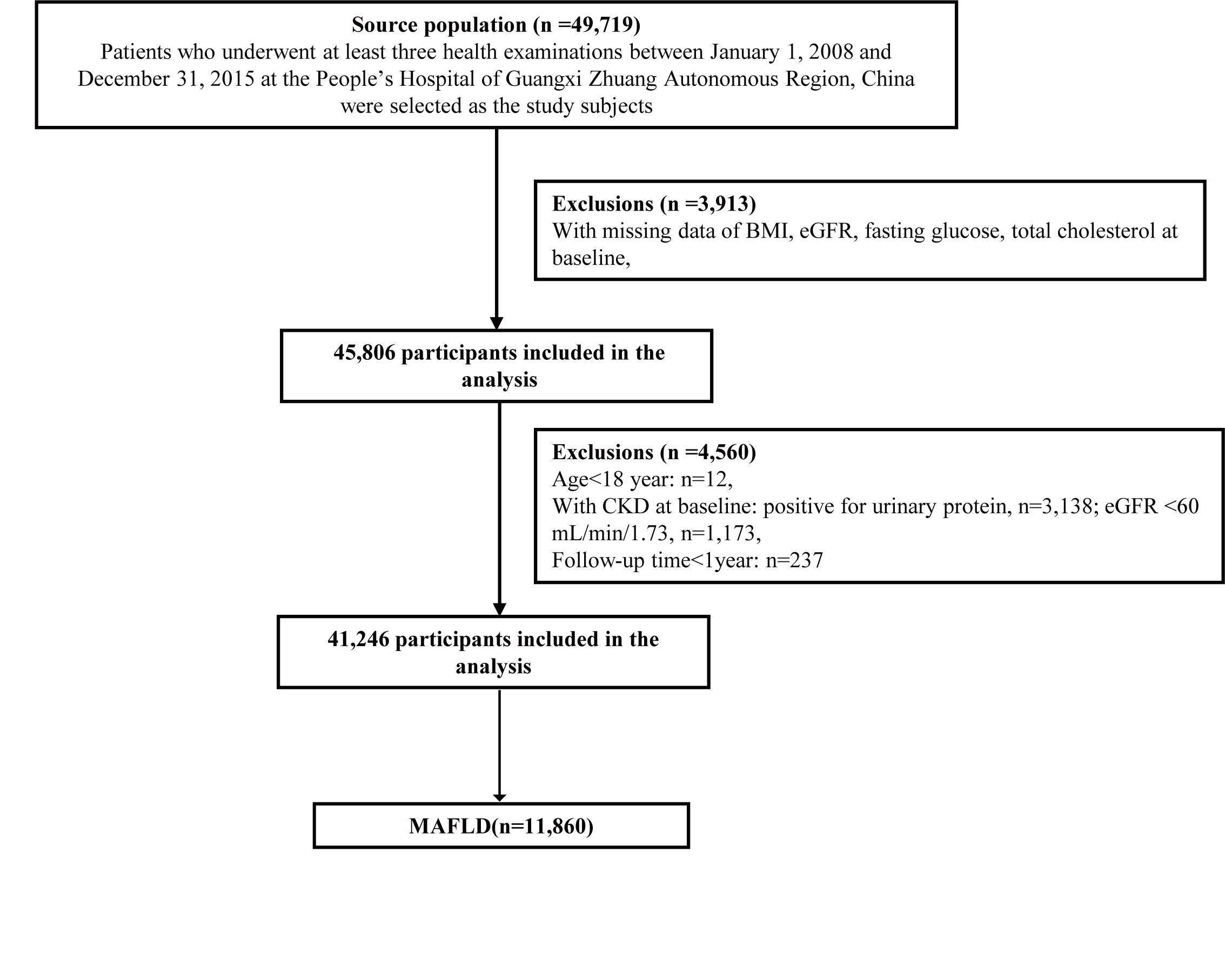

Supplement: Supplementary Figure S1 — Flow chart for the selection of participants. [file Image_1.tif]
